# Translation and Validation of the Tamil Version of the Dysphagia Handicap Index in Tamil-Speaking Patients

**DOI:** 10.7759/cureus.38652

**Published:** 2023-05-06

**Authors:** Manu Coimbatore Balakrishnan, Geetha Kishan Siddapur, Premnath Dhasaram, Nikhilesh Onnu Gopinath, Karthick Murugan, Vandhana Murugesan

**Affiliations:** 1 Otolaryngology - Head and Neck Surgery, Sri Lakshmi Narayana Institute of Medical Sciences, Puducherry, IND; 2 Community Medicine, Sri Lakshmi Narayana Institute of Medical Sciences, Puducherry, IND

**Keywords:** domain, translation and validation, covid-19, quality of life (qol), dysphagia handicap index

## Abstract

Introduction

Dysphagia is one of the general symptoms encountered in clinical practice. The impact of dysphagia can be devastating to a patient's physical condition and quality of life (QOL). To evaluate the QOL of patients with dysphagia there are numerous self-reported questionnaires. The most commonly used one such questionnaire is the Swallowing Quality-of-Life Questionnaire (SWAL-QOL). However, it is not concise and is incomplete as it does not address all the aspects of dysphagia. To overcome this, the Dysphagia Handicap Index (DHI) was developed. It focuses on the functional and emotional aspects in addition to the physical aspects of dysphagia.

Objective

To develop a Tamil version of the DHI (DHI-T) and assess its reliability, cultural adaptability and validity.

Materials and method

This cross-sectional study was conducted from May 2021 to December 2022 on 140 participants consisting of 70 dysphagia patients and 70 healthy individuals.

Results

The reliability and validity of the DHI-T were good with a high correlation between DHI-T and self-perceived severity scales of dysphagia. The mean total score in the Dysphagia group was 59.77 with the mean physical, functional and emotional scores being 23.86, 17.46 and 18.46 respectively. These scores were less compared to the Healthy group (p-value <0.01).

Conclusion

This study shows that DHI-T can be used as a reliable and valid tool to grade and study the different domains of dysphagia in our study population. Among the various causes of dysphagia studied in our population, it was noted that coronavirus disease 2019 (COVID-19)-related dysphagia patients had higher mean score in the emotional domain. To the best of our knowledge, the DHI scores for COVID-19-related dysphagia have not been done before. As the application of DHI in routine clinical practice and research is increasing, we believe this DHI-T can be of aid to Tamil-speaking patients.

## Introduction

Dysphagia is one of the general symptoms encountered in clinical practice. Dysphagia can be seen in up to 20% of the general population and has diverse aetiology, varying from neurogenic (e.g., cerebrovascular accidents or neurodegenerative diseases), head and neck tumours, infections, surgery or radiation-related [1-4]. The impact of dysphagia can be on patient's physical aspects or quality of life (QOL). Dehydration, aspiration pneumonia and malnourishment are some of the devastating physical effects of dysphagia [5,6].

To evaluate the QOL of patients with dysphagia there are numerous self-reported questionnaires. The most commonly used questionnaire is the Swallowing Quality-of-Life Questionnaire (SWAL-QOL) with high internal consistency (Cronenberg's alpha value of >0.7) [7]. It has a moderate degree of validity (Pearson's Correlation coefficient >0.40 indicating moderate correlation) [7-9]. The SWAL-QOL has 44 questions with scores for each question ranging from 1 to 5 and it can take up to 20 minutes to complete the entire SWAL-QOL. Sometimes the physician may have to assist the patient in understanding a few questions of SWAL-QOL, thereby increasing the total time to complete the questionnaire [7]. It also does not address the emotional and functional aspects of dysphagia. Hence SWAL-QOL is considered incomplete and not concise [10]. To overcome this the Dysphagia Handicap Index (DHI) was developed [10]. 

Silbergleit et al. devised the DHI and found it to be a reliable questionnaire [10]. It has a strong test-retest reliability and high internal consistency with Pearson's Correlation coefficient of 0.83 and Cronenberg's alpha value of 0.94 [10]. Furthermore, it correlates highly with self-reported dysphagia severity scores among patients, making it a valid test [10]. The routine use of DHI in clinical practice is increasing. The original DHI was devised in English and it cannot be used worldwide as many people do not know English. This has led to the DHI being translated into multiple languages such as Arabic, Persian, Hebrew and Japanese [11-14]. In India, translation and validation of DHI have been done only in the Kannada language (DHI-K) [15]. In Kannada-speaking patients with dysphagia, DHI-K has proven to be a reliable and valid tool for the assessment of QOL but cannot be used in this part of the South Indian population since the local language is Tamil. Currently, there is no DHI in Tamil. To use the DHI in our population, a validated Tamil version of the DHI is needed. Our objective was to develop a Tamil version of the DHI (DHI-T) and assess its reliability, cultural adaptability and validity. 

## Materials and methods

This cross-sectional study was conducted in the Department of Otorhinolaryngology after obtaining Institutional ethics committee approval from Sri Lakshmi Narayana Institute of Medical Sciences (IEC/C-P/20/2021).

Sample size

Total sample size was calculated to be 135 based on the formula

Z^2^_(1 − α/2)_ * σ^2^ / d^2^,

where Z^2^_(1 − α/2)_ is 3.84, σ is 7.99, d is 1.35 at 95% confidence interval [15]. A total of 140 participants were included in the study with the “Dysphagia group” labelled as Cases consisting of 70 patients and the “Healthy group” labelled as Controls consisting of 70 participants. Informed consent was obtained from all the participants.

Inclusion criteria

All Tamil-speaking patients who presented to our department as out-patient or in-patient with complaints of dysphagia from May 2021 to December 2022 were included as cases in the study. Participants without dysphagia were included in the control group. Participants who were willing to participate in the study.

Exclusion criteria

Patients with dysphagia who did not understand or speak Tamil were excluded from the study. Participants with dysphagia, previous history of chemoradiation, previous history of head and neck malignancy and stroke were excluded from the control group.

Dysphagia and healthy groups

Seventy cases included in the study were all Tamil-speaking patients with dysphagia. Seventy controls included in the study were all Tamil-speaking healthy individuals without dysphagia and were patients' attendees from the community.

Study procedure

The original version of DHI was translated into Tamil by two proficient Tamil speakers who were also proficient in English. The two separate translated versions were compiled into a single version. The translated Tamil version was reverse-translated into English version by two Tamil and English-speaking audiologists. It was compared with the original version of DHI by the authors and no major change was recommended [15-17]. The Institutional Ethics Committee approval was obtained and the DHI-T was pilot-tested on 10 patients with dysphagia and all the participants agreed that they could understand and answer the questionnaire. No further changes were made to the DHI-T and the study proceeded further by administering DHI-T to 70 patients with dysphagia and 70 controls.

All participants underwent routine clinical examination and examination of the oral cavity and oropharynx. The DHI-T questionnaire was then administered to the participants and their responses were recorded after obtaining written informed consent. The study was conducted during coronavirus disease 2019 (COVID-19) pandemic and so elective functional endoscopic evaluation of swallowing (FEES) and video laryngoscopy examination (VLE) were not done fearing cross infection to patients and exposure to the doctor [18,19].

The DHI contains 25 items, divided into physical, functional and emotional domains. Nine items are included to evaluate the patient's perception of the physical discomfort due to dysphagia (physical QOL), nine items to evaluate the patient's perception of the effect of dysphagia on day-to-day activities (functional QOL) and seven items to evaluate the affective response of the patient to one's dysphagia (emotional QOL). The total score for the DHI will range from zero to 100 with each item given a score of 0 (never) or 2 (sometimes) or 4 (always). High scores indicate low QOL [10]. After completing the DHI questionnaire the patients were requested to rate the severity of one's dysphagia on a Likert scale of 1 to 7 with 1 being normal and 7 being severe.

Statistical tools

For the test-retest reliability testing, 10% of the participants were made to fill out the same questionnaire after two weeks. After data collection, the data were analysed using SPSS version 16.0, 2007 (SPSS Inc., Chicago, IL, USA). Intraclass correlation coefficient was used to analyse test-retest reliability of the DHI-T questionnaire. In the dysphagia group for the self-perceived severity assessment, the test-retest reliability was measured using Cohen's kappa agreement. DHI-T scores of patients were compared with DHI-T scores of controls for establishing validity. All the statistical tests were considered statistically significant at a p-value of less than or equal to 0.05.

## Results

Table 1 depicts the different causes of dysphagia in our study population. The mean age±S.D. in our study was 38.66±14.89 years with the minimum and maximum ages being 22 and 76 years respectively. Male participants constituted 76 (54%) and female participants 64 (46%) in our study. Table 2 shows the intraclass correlation coefficient used to analyse test-retest reliability of the DHI-T questionnaire. In the dysphagia group for the test-retest reliability of the patient's self-perceived severity scale, Cohen's kappa agreement was 0.88 indicating almost perfect agreement.

**Table 1:** Different causes of dysphagia in our study population

Dysphagia etiology N=70	n (%)
Neurogenic cause	22 (31%)
Head and neck malignancy	21 (30%)
Oesophageal cause	6 (9%)
COVID-19 related dysphagia	20 (29%)
Other/Unknown	1 (1%)

**Table 2:** Test–retest reliability of the DHI-T questionnaire DHI-T: Dysphagia Handicap Index Tamil

Domains of DHI-T	Test-retest reliability Intraclass Correlation Coefficient
Physical	0.99
Functional	0.99
Emotional	0.98
Total	0.99

Table 3 shows the distribution of DHI-T scores according to self-perceived dysphagia severity. The Spearman correlation coefficient was used between the subscales of DHI-T and the self-perceived severity rating of the dysphagia group and was found to be very high (Table 4).

**Table 3:** Distribution of DHI scores according to self-perceived dysphagia severity Values in Mean (S.D.); DHI: Dysphagia Handicap Index

Domain (N=70)	Normal (n=7)	Moderate (n=43)	Severe (n=20)
Physical	12.57 (4.12)	22.60 (5.62)	30.50 (2.50)
Functional	8.57 (4.28)	14.42 (7.55)	27.10 (2.79)
Emotional	5.43 (3.41)	19.86 (7.40)	20.0 (4.0)
Total	26.36 (9.43)	56.88 (9.97)	77.60 (6.21)

**Table 4:** Correlation between self-perceived severity and subscales of DHI-T DHI-T: Dysphagia Handicap Index Tamil

Domain of DHI-T	Spearman correlation for self-perceived severity
Physical	0.95
Functional	0.94
Emotional	0.93
Total	0.87

Mann Whitney test was used to compare the healthy group with the Dysphagia group in terms of the total DHI-T scores and the subscales of DHI-T. The scores for both total and subscales of DHI-T were significantly lower for the Healthy group than the Dysphagia group and this was found to be statistically significant (Table 5).

**Table 5:** Comparison of DHI-T scores between Dysphagia group and Healthy group Values in Mean (S.D.); *Mann-Whitney Test; DHI-T: Dysphagia Hanidcap Index Tamil

Domains	Dysphagia group	Healthy group	p value*
Physical	23.86 (7.01)	1.06 (1.62)	< 0.01
Functional	17.46 (8.89)	0.29 (0.98)	< 0.01
Emotional	18.46 (7.61)	0.17 (0.56)	< 0.01
Total	59.77 (16.97)	1.51 (2.77)	< 0.01

Table 6 depicts the distribution of scores for the DHI-T subscales in the Dysphagia group according to different causes. The mean of the emotional component of DHI-T was higher among participants with COVID-19-related dysphagia compared to the participants with dysphagia of other causes (26.30 vs 15.36). This finding was found to be statistically significant (Table 7).

**Table 6:** Distribution of DHI-T scores for different causes of dysphagia (N=70) Values in Mean (S.D.); DHI-T: Dysphagia Handicap Index Tamil

Causes of Dysphagia (n)	Physical	Functional	Emotional	Total DHI-T
Neurogenic cause (22)	27.73 (4.91)	24.00 (5.65)	16.91 (6.13)	68.64 (15.50)
Head and Neck malignancy (21)	27.52 (5.02)	21.33 (5.07)	15.71 (4.83)	64.57 (13.33)
Oesophageal causes (6)	12.80 (5.02)	10.00 (2.00)	10.40 (5.17)	33.20 (12.13)
COVID-19 related Dysphagia (20)	18.70 (4.07)	8.40 (6.38)	26.20 (5.31)	53.30 (9.91)
Others/ Unknown (1)	22.00 (14.14)	14.00 (16.97)	7.00 (9.89)	43.00 (41.01)

**Table 7:** Comparison of DHI-T scores of different domains with causes of dysphagia (N=70) Values in Mean (S.D.); *Neurogenic, Oesophageal, Head and Neck Malignancies, Unknown; # Mann Whitney Test; DHI-T: Dysphagia Handicap Index Tamil

Causes of Dysphagia (n)	Physical	Functional	Emotional	Total DHI-T
COVID-19 related Dysphagia (20)	18.70 (4.07)	8.40 (6.38)	26.20 (5.31)	53.30 (9.91)
Dysphagia due to other Causes* (50)	25.92 (6.89)	21.08 (6.99)	15.36 (6.03)	62.36 (18.54)
p Value^#^	<0.01	<0.01	<0.01	<0.01

## Discussion

The physical effects of dysphagia range from malnutrition and dehydration, to aspiration pneumonia and asphyxia [5,6]. Some of the self-reported questionnaires for assessing the QOL of dysphagia patients are specific to particular diseases such as the Dysphagia Goal Handicap. It focuses on patients with oesophageal phase dysphagia. The M.D. Anderson Dysphagia Inventory focuses on patients with head and neck cancers and swallowing disorders. The SWAL-QOL is a generic one and is applicable to any disease [20-22]. The DHI is highly concise, however, the original English version cannot be applied to the worldwide population necessitating the translation of DHI to regional languages, i.e. Tamil in our case. The self-reported QOL of patients with dysphagia is needed for a holistic approach to treatment and to focus on the specific component for therapy. This holistic approach not only focuses on the physiological aspect of dysphagia but also the psychological and social aspects too. 

Our study focused on the translation and validation of the DHI in Tamil-speaking patients. In our study, neurogenic cause (31%), head and neck malignancy (30%) and COVID-19-related dysphagia (29%) contributed to the major portion of cases. In the original DHI study by Silbergleit et al., neurological conditions (52%) were followed by unknown aetiology (23%) as the major portion [10]. DHI-T had good test-retest reliability, correlation with the self-perceived severity scale of dysphagia and validity. This was similar to the original DHI and other translated versions of DHI by Oda et al. in Japanese, Shapira et al. in Hebrew and Krishnamurthy et al. in Kannada [10,13-15]. The total mean score for DHI-T was 59.77 (16.97). The total mean score in the original DHI by Silbergleit et al. was 27.33 (21.28). This was in contrast to our study and could be possibly explained by the fact that patients with mild dysphagia contributed to the major part of that study [10]. The total mean scores in Hebrew DHI and Persian DHI were 38.44 (24.39) and 32.14 (25.32) respectively [11,13]. Similar to our finding was the case in DHI-K by Krishnamurthy et al. with total mean scores of 77.72 (21.25) [15].

Among the different domains in DHI-T, the mean score of the physical domain was more in our study i.e. 23.86 (7.01). This was the similar findings noted in Original DHI, Hebrew DHI and Persian DHI [10,11,13]. However, in contrast to all these studies, in DHI-K the mean score of the functional domain was more [15]. Table 8 depicts the comparison of DHI scores across different domains in different studies. 

**Table 8:** Comparison of different DHI scores across different domains in different studies *- values in mean (S.D.); DHI-Dysphagia Handicap Index; DHI-T: Dysphagia Handicap Index Tamil; DHI-K: Kannada

Our study DHI-T* (N=70)			
Domain	Normal (n=7)	Mild (n=43)	Moderate/Severe (n=20)
Physical	12.57 (4.12)	22.60 (5.62)	30.50 (2.50)
Functional	8.57 (4.28)	14.42 (7.55)	27.10 (2.79)
Emotional	5.43 (3.41)	19.86 (7.40)	20.0 (4.0)
DHI*[10] (N=60)			
Domain	Normal (n=19)	Mild (n=29)	Moderate/Severe (n=12)
Physical	10.32 (6.16)	13.03 (6.71)	16.50 (7.34)
Functional	7.58 (10.10)	9.52 (8.03)	20.33 (11.81)
Emotional	6.32 (7.25)	5.17 (5.72)	13 (10.25)
DHI-K*[15] (N=88)			
Domain	Normal (n=1)	Mild (n=10)	Moderate/Severe (n=77)
Physical	28 (0)	34	32.82 (2.55)
Functional	29 (7.07)	34	33.29 (1.86)
Emotional	25 (1.41)	22	26.47 (1.66)

In our study, it was also noted that among COVID-19-related dysphagia patients, the mean score was more in the emotional domain. To the best of our knowledge, the DHI scores for COVID-19-related dysphagia have not been done before and we believe this study could shed light on the same and necessitates the need for further studies on COVID-19-related dysphagia focusing on emotional domains. Patients in mechanical ventilation with oral intubation can have a risk of developing swallowing difficulties [23-26]. A high proportion of patients who recovered from COVID-19 experienced some form of dysphagia. Some of the factors which may contribute to the development of oropharyngeal dysphagia in COVID-19-recovered patients are age > 65 years, neurologic symptoms and muscle weakness due to severe acute respiratory syndrome coronavirus 2 (SARS-CoV-2), cognitive deterioration, prolonged ICU care, stress and poor QOL during quarantine [27-30].

The strength of the study is that the sample size is considerable and no prior study has been conducted in our population. It also highlighted that the emotional domain can be affected more in COVID-19-related dysphagia. However, the above statement needs further validation with a large sample size in COVID-19 recovered patients. The limitations of the study are the inability to support the DHI scores with other investigations like FEES and VLE. However, the authors have supplemented the DHI-T scores with patient-perceived self-reported severity scales. The authors can provide the DHI-T upon request. 

## Conclusions

The authors conclude from the above study that DHI-T can be used as a reliable and valid tool to grade dysphagia in Tamil speaking population. DHI-T can be used to study the different domains of dysphagia for the holistic treatment of dysphagia. As the application of DHI is increasing, we believe this DHI-T can be of aid in routine clinical practice and research in Tamil-speaking patients.
